# Spatiotemporal Variation and Pollution Assessment of Pb/Zn from Smelting Activities in China

**DOI:** 10.3390/ijerph17061968

**Published:** 2020-03-17

**Authors:** Zhenfeng Zang, Yonghua Li, Hairong Li, Zhaohui Guo, Ru Zhang

**Affiliations:** 1 Key Laboratory of Land Surface Pattern and Simulation, Institute of Geographic Sciences and Natural Resources Research, Chinese Academy of Sciences, Beijing 100101, China; zangfengaa@163.com (Z.Z.); lihr@igsnrr.ac.cn (H.L.); zhangr.17b@igsnrr.ac.cn (R.Z.); 2 University of Chinese Academy of Sciences, Beijing 100049, China; 3 Institute of Environmental Engineering, School of Metallurgy and Environment, Central South University, Changsha 410083, China; zhguo@csu.edu.cn

**Keywords:** spatiotemporal variation, Pb/Zn smelting, environmental release, soil, heavy metal pollution, potential ecological risk assessment

## Abstract

Nonferrous smelting is an important source of heavy metals in soil, which causes different degrees of soil pollution, especially in lead–zinc (Pb/Zn) smelting areas. Based on the Pb/Zn environmental release during the Pb/Zn mineral smelting processes in 31 Chinese provinces from the period 2000 to 2015, the study analyzed the spatiotemporal variations in Pb/Zn environmental release at the national level and then evaluated the degree of soil pollution and potential ecological risk based on the heavy metal content in soil from Pb/Zn smelting areas. The results showed that from the period 2000 to 2015, the Pb release had a discrete trend, transferring from the middle east to the west, and the provinces with higher Pb releases were Henan Province, Yunnan Province, and Hunan Province. However, the Zn release showed a tendency towards spatial aggregation, and the release center of this heavy metal presented a shifting trend from south to north; additionally, the release of Zn was highest in Hunan Province. The pollution index analyses indicated that Cd, Pb, Cu, and Zn in Zhuzhou and Huludao all reached extremely polluted levels, while Tongguan was at a safe level. In Gejiu, Mianxian, Fengxian, Zhuzhou, Huludao, and Shaoguan, there were very high potential ecological risks, with Cd having the highest potential ecological risk in most smelting areas, followed by Pb.

## 1. Introduction

The rapid development of industrialization resulted in many environmental problems; soil heavy metal pollution has especially caused worldwide concern due to their persistence, toxicity, and harmful effects on plants and human health [1,2,3,4,5,6]. Nonferrous metal smelting is an important source of heavy metals in soil [7,8,9,10]. Heavy metals enter the soil mainly through atmospheric deposition, sewage irrigation, and slag percolation, resulting in various levels of heavy metal contamination in cultivated land around a smelter [7,11,12,13]. Many studies have shown that heavy metal pollution in soil around nonferrous metal smelting areas is very serious [4,14,15,16]. The Pb/Zn smelting is an important source of heavy metals in soil [8,9,11,16,17]. During the smelting process, some associated heavy metal elements, such as Cd, Cu, and As, are also released into the surrounding environment [1,18,19,20]. 

China is a country with abundant Pb and Zn resources, and its Pb and Zn mineral reserves are second only to Australia in the world, and Pb/Zn mines are widely distributed in China [21]. With the growing demand for Pb and Zn resources in China, the environmental problems arising from the Pb/Zn smelting process cannot be ignored. During the smelting of Pb/Zn minerals, a large amount of waste water, waste gas and solid waste is generated; if improperly treated or discharged, it causes soil heavy metal pollution, and ultimately results in harm to human health through the food chain [6,14,22,23,24]. At present, studies on the environmental release of Pb/Zn during smelting in China are insufficient, and the environmental release and distribution of Pb/Zn at the provincial level is unclear.

At present, the research at home and abroad in Pb/Zn smelting areas mainly focuses on pollution and risk assessment of soil and crops around smelting areas, and the research areas are mostly small or medium-sized [4,14,16,24,25,26,27]. There is a lack of comprehensive evaluation of the differences in pollution degree and potential ecological risk in different regions.

It is important to understand the spatiotemporal variation in the environmental release of Pb/Zn and the difference in pollution degree and potential ecological risk in different regions. For this purpose, we carried out a study to (a) analyze the spatiotemporal variation in Pb/Zn environmental release and (b) explore the difference in soil heavy metal pollution and potential ecological risk in different Pb/Zn smelting areas.

## 2. Materials and Methods

### 2.1. Materials

#### 2.1.1. Pb/Zn Environment Release during Smelting

Data on the Pb/Zn production and smelting recovery of minerals in provinces came from the yearbook of nonferrous metals industry of China (2001–2016). In this study, 31 provincial administrative regions were included (excluding Hong Kong, Macao and Taiwan). Our data on the release of Pb/Zn to the environment are at provincial scale. In provinces with no available data, a value of 0 was used.

The calculation formula of Pb/Zn release in the smelting process is as Equation (1) [28].

(1) $$S_{r}=M_{\mathrm{ep}}\times \left(1-S_{\mathrm{ar}}\right)$$

where S_r_ is the smelting release, M_ep_ is the mineral metal production, and S_ar_ is smelting average recovery rate. The smelting release refers to the release from the smelting process through mediums such as atmosphere, water and solid.

#### 2.1.2. The Content of Heavy Metals in Soil from Pb/Zn Smelting Areas

In Pb/Zn smelting areas, the concentrations of heavy metals in the soil were collected from the literature; we used ‘Web of Science’ and ‘China’s National Knowledge Infrastructure project (CNKI)’, and the subject terms “Pb/Zn smelting, heavy metal” and “smelting plant, soil heavy metal” to select the literature that met the requirements. The soil samples in literates were topsoil (0~20 cm), and the digestion method of the samples was consistent with the China national standards (GB15618–1995). The quality control of the samples test was also less than 10%. We finally screened a total of 21 references, which have been published since 2005, on heavy metals in soil from Pb/Zn smelting areas.

### 2.2. Methods

#### 2.2.1. Spatial Autocorrelation Analysis

Spatial autocorrelation analysis is mainly applied to detect whether there is an aggregation trend between a spatial unit and its adjacent spatial units [29]. The global Moran’s index (GMI) is a commonly used indicator for evaluating spatial autocorrelation statistics. When the GMI increases, the spatial correlation increases, and the variables show spatial aggregation. In contrast, lower GMI values indicate that the correlation has become weaker, and the variables appear as spatially discrete trends. This research utilizes spatial autocorrelation analysis to study the overall distribution of Pb/Zn environmental release in China; the calculation formula is as Equation (2).

(2) $$\mathrm{GMI}=\frac{\sum_{i=1}^{n}\sum_{j=1}^{n}W_{\mathrm{ij}}\left(x_{i}-\bar{x}\right)\left(x_{j}-\bar{x}\right)}{S^{2}\sum_{i=1}^{n}\sum_{j=1}^{n}W_{\mathrm{ij}}}$$

where n is the number of provincial units, x_i_ and x_j_ are the Pb and Zn environmental releases for provincial units i and j, respectively, W_ij_ is the standardized spatial weight of provincial units i and j. The GMI value is between −1 and 1. If the GMI value is positive, this indicates that the Pb/Zn release has a significant aggregation state in space, and if it is negative, this indicates that the Pb/Zn release shows a discrete situation in the spatial distribution. To identify the significance of the GMI, the Z test is applied, and the calculation formula is as Equation (3).

(3) $$Z\left(I\right)=\frac{1-E\left(I\right)}{\sqrt{V\left(I\right)}}$$

where Z(I) is the Z test value of the GMI, and E(I) and V(I) are the mathematical expectation and variance of the GMI, respectively.

#### 2.2.2. Evaluation of Soil Heavy Metal Pollution

To evaluate the soil heavy metal pollution in Pb/Zn smelting areas, the single pollution index is applied [30]. The calculated formula is as Equation (4).

(4) $$P_{i}=\frac{C_{i}}{S_{i}}$$

where P_i_, C_i_, and S_i_ represent the pollution index, measured concentration, and evaluation standard value of heavy metal i, respectively. In this study, the S_i_ values are selected from the risk screening values in “Soil environmental quality—Risk control standard for soil contamination of agricultural land”. The grading standard of the single pollution index is as follows: P_i_ ≤ 1, clean; 1 < P_i_ ≤ 2, lightly polluted; 2 < P_i_ ≤ 3, moderately polluted; P_i_ > 3, highly polluted.

#### 2.2.3. Potential Ecological Risk Assessment

The potential ecological risk index method is applied to evaluate the ecological risk of heavy metals in smelting areas. The method was proposed by Hakanson in 1980 [31], and the results can reflect the potential ecological risk degree of heavy metals in the soil to the environment. The calculation formula is Equation (5).

(5) $$\mathrm{RI}=\sum_{i=1}^{4}E_{i}=\overset{4}{\underset{i=1}{\sum }}T_{i}\times \frac{C_{i}}{C_{b}}$$

where RI is the comprehensive potential ecological risk index of various heavy metals; E_i_ is the potential ecological risk index of heavy metal i in the soil; T_i_ is the toxicity response coefficient of heavy metal i; the toxicity response coefficients of Cd, Pb, Cu, and Zn are 30, 5, 5, 1, respectively; C_i_ and C_b_ are the measured concentration and the background value of soil heavy metal i, respectively (mg/kg). In this study, the corresponding soil background values are selected according to the soil region. The classification criteria for potential ecological risks are as follows: E_i_ < 40, RI < 150, indicates a low potential ecological risk; 40 ≤ E_i_ < 80, 150 ≤ RI < 300, means medium potential ecological risk; 80 ≤ E_i_ < 160, 300 ≤ RI < 600, represents considerate potential ecological risk; 160 ≤ E_i_ < 320, 600 ≤ RI < 1200, denotes high potential ecological risk; and E_i_ ≥ 320, RI ≥ 1200, signifies very high potential ecological risk [25,31].

## 3. Results and Discussion

### 3.1. Spatial and Temporal Variations in Pb/Zn Eenvironmental Release

#### 3.1.1. Temporal Variation in Pb/Zn Environmental Release

**Figure 1:**
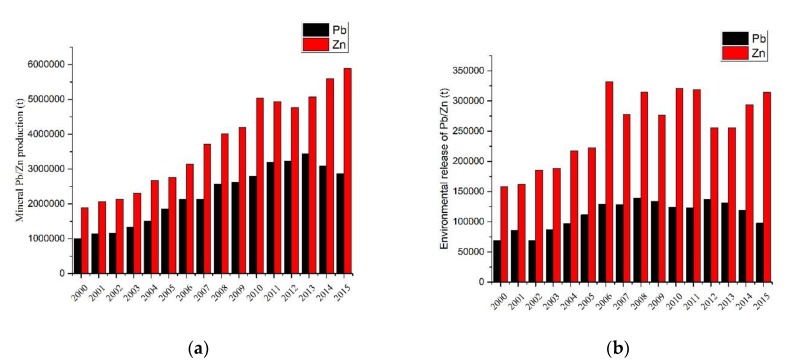
China’s mineral Pb/Zn production and environmental release from the period 2000 to 2015. (**a**) mineral Pb/Zn production; (**b**) environmental release of Pb/Zn.

The mineral Pb/Zn production and environmental release from the period 2000 to 2015 is shown in Figure 1; specifically, the production of Pb increased from the period 2000 to 2013, with a maximum production of 3.44 × 10^6^ ton in 2013 and a decrease after 2014. Overall, the production of Zn was increasing, and the maximum output was 5.88 × 10^6^ ton in 2015. The production of Zn was higher than that of Pb.

The increase in mineral Pb/Zn production is mainly due to the growing demand for Pb/Zn in recent years in China [21,28], although the production of Pb/Zn is vast, and several tons were also imported from other counties. In recent years, the decline in Pb production has been mainly caused by price factors, environmental protection policies and other factors [21]. With the increase in Pb/Zn production, the release of Pb/Zn to the environment during smelting cannot be ignored.

The amount of Pb/Zn released into the environment during the smelting process is shown in Figure 1b; specifically, the environmental release of Pb first increased and then decreased, and the maximum amount of Pb released was 1.39 × 10^5^ ton in 2008. The amount of Zn released first increased and then alternately increased and decreased, and the maximum amount of Zn released was 3.32 × 10^5^ ton in 2006. The release of Pb/Zn to the environment is not always consistent with the level of production, which is mainly due to strict environmental policies, emission reduction measures and technological improvements, resulting in a reduction in Pb/Zn release [21,28,30,32].

#### 3.1.2. Spatiotemporal Variation in Pb/Zn Environmental Release

**Figure 2:**
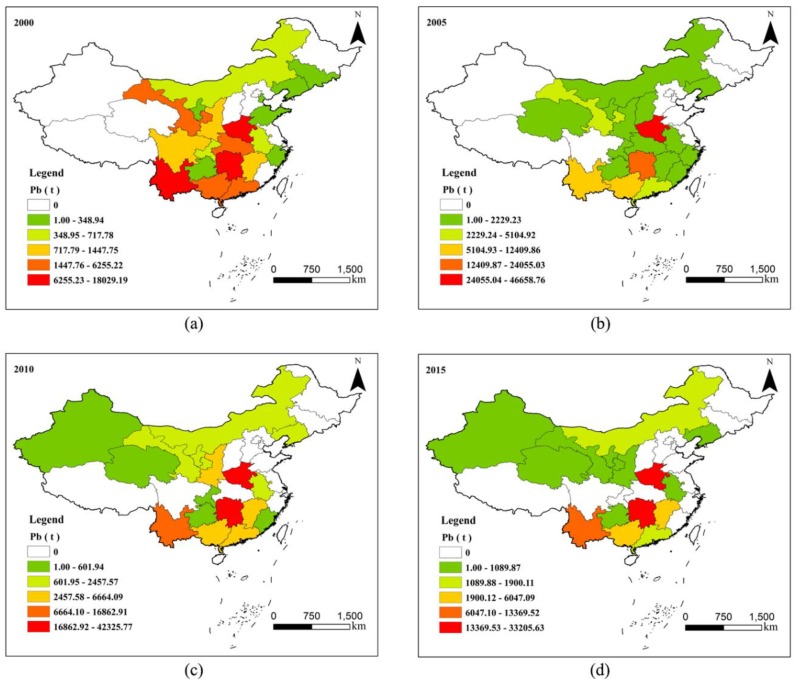
Spatial distribution characteristics of Pb environmental release in China. (**a**) spatial distribution of Pb in 2000; (**b**) spatial distribution of Pb in 2005; (**c**) spatial distribution of Pb in 2010; (**d**) spatial distribution of Pb in 2015.

Based on the release of Pb to the environment at the provincial scale, and through the function of color classification in ArcGIS 10.3, the provinces with different release levels are shown in different colours. The spatial distribution maps for Pb in 2000, 2005, 2010, and 2015 are shown in Figure 2. The environmental release of Pb indicated an extremely unbalanced phenomenon between regions in China.

From Figure 2a, low and sub-low areas were mainly distributed in the north and east of China, and high and sub-high areas were mainly located in the middle and south, especially in Henan Province, Hunan Province and Yunnan Province. In 2005, the low value areas were mainly located in the northern and south-central regions, and the high value area was in Henan Province. In some provinces, the emission had reduced to zero, such as in Shandong Province, Tianjin, Jilin Province, and Sichuan Province; however, some additional provinces were added, e.g., Qinghai Province, Fujian Province, and Shanxi Province. In 2010, the low and sub-low areas were mainly distributed in the north; the high value areas were distributed in Henan Province and Hunan Province; and the major change in emission provinces was that Shanxi Province, Hubei Province, and Zhejiang Province became no-release areas, while Xinjiang Autonomous Region was added. In 2015, apart from Fujian Province and Chongqing becoming no-release areas, the distribution pattern was similar to that in 2010, in which the low and sub-low areas were mainly distributed in the north, and the high value area was distributed in Henan Province and Hunan Province.

From the period 2000 to 2015, the number of provinces with Pb releases was reduced from 20 to 15; the reduced area was mainly distributed in the central and eastern regions, while the added area was mainly located in the western region. The area of released Pb showed a tendency of transferring from the middle east to the west, which was mainly due to the distribution of mine Pb resources and the impact of environmental protection policies [21,32].

**Figure 3:**
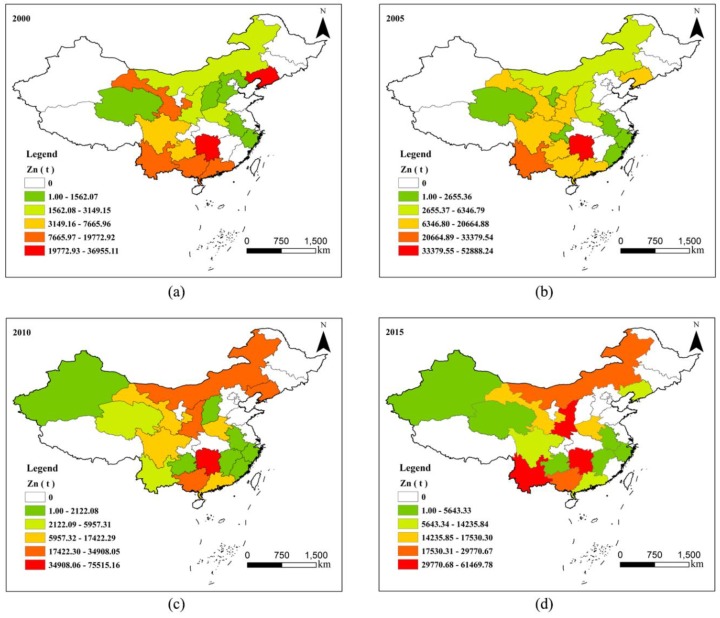
Spatial distribution characteristics of Zn environmental release in China. (**a**) spatial distribution of Zn in 2000; (**b**) spatial distribution of Zn in 2005; (**c**) spatial distribution of Zn in 2010; (**d**) spatial distribution of Zn in 2015.

According to the release of Zn to the environment at the provincial scale and through the function of color classification in ArcGIS 10.3, the provinces with different release levels are shown in different colours. The spatial distribution maps of Zn in 2000, 2005, 2010, and 2015 are displayed in Figure 3. The spatial distribution of Zn also showed obvious regional differences.

In 2000, the low and sub-low value areas were mainly distributed in the north, the high and sub-high value areas were mainly located in the south, and Hunan Province had the highest release. In 2005, the distribution pattern was similar to that in 2000, but three new provinces were added to the distribution area, namely, Fujian Province, Chongqing, and Ningxia Hui Autonomous Region, and Hebei Province was removed. The low and sub-low value regions were mainly in the north and east, and the high and sub-high value areas were in Hunan Province and Yunnan Province. In 2010, there were some changes in the distribution of provinces, including the addition of Xinjiang Uygur Automous Region and Jiangxi Province and the reduction in Ningxia Hui Autonomous Region and Chongqing. The sub-high region was transferred from the south to north compared to the distribution in 2005, and the high value region was in Hunan Province. In 2015, the distribution provinces were reduced by two provinces compared with 2010, namely, Shanxi Province and Fujian Province; the low areas were located in the northwest and east; the high and sub-high regions were in the north and south; and the high value regions were in Hunan Province, Yunnan Province, and Shaanxi Province.

From 2000 to 2015, although there were some changes in areal distribution, the total number of Zn release provinces did not change. Among them, Hunan Province released the most. The release center of Zn showed a trend of shifting from south to north, which was mainly due to the gradual exploitation of Zn resources from the south to the north and environmental policy [21,32].

### 3.2. Spatial Autocorrelation Analysis of Pb/Zn Environmental Release

As shown in Table 1, for Pb environmental release, in 2000, 2005, 2010, and 2015, the Moran’s I values were 0.0651, −0.0327, −0.0109, and −0.0118, respectively. The Z-value < 2.58, which indicated that the correlation was poor. Only in 2000 was there a certain accumulation tendency of Pb release in provincial unit. In 2005, 2010, 2015, the amount of Pb release showed a discrete trend.

As Table 1 shows, for Zn environmental release in 2000, 2005, 2010, and 2015, the Moran’s I values were 0.0686, 0.1156, 0.0152, and 0.0455, respectively, which were all greater than 0, and there was a certain tendency of spatial aggregation of Zn. The spatial correlation was greatest in 2005.

### 3.3. Pollution Assessment of Soil Heavy mMetals in Pb/Zn Smelting Areas

To investigate the pollution degree of heavy metals in soil around the Pb/Zn smelters, data on soil heavy metal content (Cd, Pb, Cu, and Zn) in 21 smelting areas from nine provinces were collected and analyzed. The content of heavy metals in the soil is displayed in Table 2.

#### 3.3.1. Content of Heavy Metals in Soil

As shown in Table 2, the concentration of Cd was higher in Gejiu, Hezhang, Mianxian, Fengxian, Zhuzhou, and Huludao, with concentrations of 63.50, 43.00, 39.74, 33.79, 108.18, and 60.94 mg/kg, respectively, while the concentration in Tongguan was low, with a value of 0.26 mg/kg. The concentration of Cd is high in the soil from the smelting areas, mainly because Cd is often associated with Pb/Zn ore. During Pb/Zn smelting, Cd was released into the surrounding environment through waste gas, waste water and other forms [51,52]. The concentrations of Pb in Gejiu, Hezhang, Baiyin ^b^, Zhuzhou, and Shaoguan were 3347.50, 9000.00, 3510.00, 7621.86, and 1185.66 mg/kg, respectively, which were higher than those in other smelting areas. The concentrations of Pb in Fengxiang and Jiaozuo were 53.50 and 42.33 mg/kg, respectively, which were lower than those in other areas, mainly due to the difference in smelting amount and smelting time [25,47]. The concentrations of Cu in Baiyin ^a^, Baiyin ^b^, Zhuzhou, and Huludao were 330.81, 426.00, 261.41, and 247.80 mg/kg, respectively, but they were lower in Lancang and Jiaozuo, with values of 14.79 and 15.98mg/kg, respectively. The concentrations of Zn in Huize, Gejiu, Hezhang, and Huludao were 1688.00, 2974.30, 11,000.00, and 4084.00 mg/kg, respectively, and the lower values were in Tongguan and Jiaozuo, with 68.60 and 67.77 mg/kg, respectively. The concentrations of heavy metals in different regions displayed great differences, mainly due to the smelting time, smelting amount, types of associated metals and other mineral production activities in the region [24,25,37].

#### 3.3.2. Pollution Assessment of Heavy Metals in Soil

**Figure 4:**
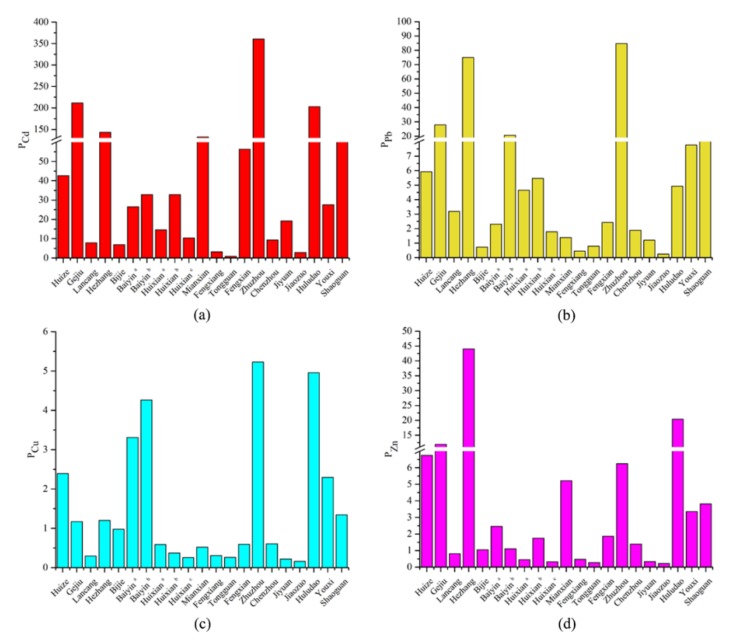
Single pollution index of heavy metal in soil. (**a**) pollution index of Cd; (**b**) pollution index of Pb; (**c**) pollution index of Cu; (**d**) pollution index of Zn.

According to the concentration of heavy metals in soil, the risk screening values in smelting areas and Equation (4), the single pollution index is shown in Figure 4.

As shown in Figure 4, the single pollution indices of Cd, Pb, Cu, and Zn varied greatly in different regions. For Cd, the single pollution index was 0.87 only in Tongguan, which indicated that the soil was clean. In Jiaozuo, the value was 2.83, reaching a moderately polluted level. In other smelting areas, the single pollution indices were all greater than 3, reaching a highly polluted level. In Gejiu, Hezhang, Mianxian, Zhuzhou, and Huludao, the values reached 211.67, 143.33, 132.47, 360.60, and 203.13, respectively, and the pollution level was quite serious. It can be seen from the degree of Cd pollution in the Pb/Zn smelting areas that Cd pollution is a common problem and is very serious in some areas. For Pb, the single pollution indices in Bijie, Fengxiang, Tongguan, and Jiaozuo were 0.73, 0.45, 0.79, and 0.25, respectively, which indicated that the soil in these regions was at a safe level. However, in other areas, the single pollution indices all exceeded 1, especially in Gejiu, Hezhang, Baiyin ^b^, Zhuzhou, and Shaoguan, which were 27.90, 75.00, 20.65, 84.69, and 16.94, respectively, and the pollution level was extremely high. For Cu, the single pollution indices in Baiyin ^a^, Baiyin ^b^, Zhuzhou, and Huludao were 3.31, 4.26, 5.23, and 4.96, respectively, which were all above 3, and reached high pollution levels. The single pollution indices in Huize and Youxi were 2.39 and 2.30, respectively, which indicated moderate pollution. The single pollution indices in Gejiu, Hezhang, and Shaoguan were 1~2, and the pollution level was slightly polluted. The single pollution indices of Cu in other areas were less than 1, and the soil was at a clean level. For Zn, the single pollution indices in Hezhang and Huludao were 44.00 and 20.42, respectively, and the lowest was in Jiaozuo, which was 0.23. The single pollution indices of Cd, Pb, Cu, and Zn in Zhuzhou and Huludao were all highly polluted; one reason for this was that the smelting in Zhuzhou and Huludao has existed for several years, and another was that smelting had caused serious pollution to local soil [44,48]. The single pollution indices of Cd, Pb, Cu, and Zn in Tongguan were all below 1, which indicates a safe level and is mainly due to the small scale of the smelter and the short smelting time [32].

### 3.4. Potential Ecological Risk Assessment

**Figure 5:**
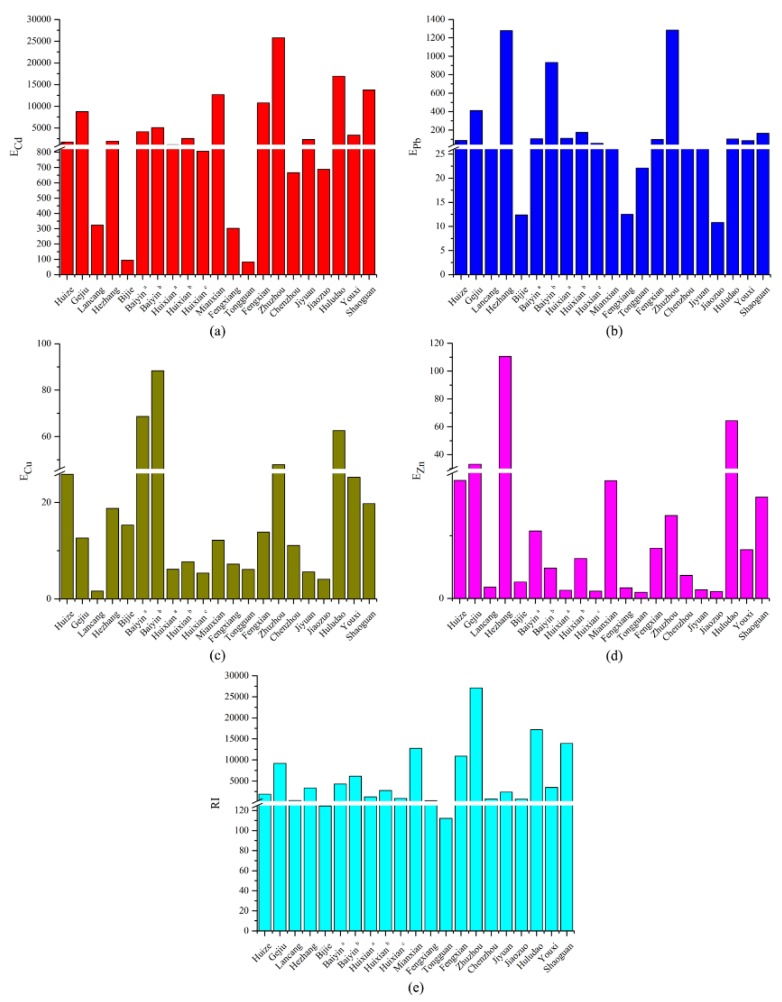
The potential ecological index of heavy metal in soil. (**a**) potential ecological risk index of Cd; (**b**) potential ecological risk index of Pb; (**c**) potential ecological risk index of Cu; (**d**) potential ecological risk index of Zn; (**e**) comprehensive potential ecological risk index.

Based on Equation (5), the potential ecological risk index is shown in Figure 5.

The potential ecological risk of Cd in Bijie and Tongguan was considerate; it was high in Fengxiang and very high in all other areas, especially in Mianxian, Fengxian, Zhuzhou, Huludao, and Shaoguan. The potential ecological risk exceeded 10,000, and the content of Cd in the soil far exceeded the respective soil background values. There were great potential ecological risks in these areas.

The potential ecological risk of Pb in Gejiu, Hezhang, Baiyin ^b^, and Zhuzhou were very high, and the values in Huixian ^b^ and Shaoguan were high, indicating that the Pb pollution was serious in these areas and may cause serious ecological harm. In other regions, the potential ecological risk was considerably lower.

The potential ecological risk level of Cu in the Pb/Zn mining area was generally low, except for the ecological risk of Cu in Baiyin ^b^, which was 88.38, reaching a considerable potential ecological risk. In Baiyin ^a^, Zhuzhou, and Huludao, the risk was moderate, and the potential ecological risk in other areas was low.

The potential ecological risk of Zn in Hezhang was considerable. In Huludao, the risk was medium, and the risk in other regions was low, indicating that the potential ecological risk level of Zn in the Pb/Zn smelting area was generally low and that the risk of causing ecological damage was small.

In general, the potential ecological risk was extremely high in Gejiu, Mianxian, Fengxian, Zhuzhou, Huludao, and Shaoguan; among them, Cd had the largest contribution, followed by Pb, and the contribution of Cu and Zn was relatively low.

## 4. Conclusions

This study analyses the spatiotemporal variation in the environmental release of Pb/Zn and the differences in soil heavy metal pollution and potential ecological risks in different Pb/Zn smelting areas. Overall, the environmental release of Pb first increased and then decreased, and the amount of Zn first increased and then decreased. However, the release of Pb/Zn to the environment was not always consistent with production. From the spatiotemporal variation in the release of Pb/Zn, the environmental release of Pb/Zn showed an extremely unbalanced phenomenon between regions in China. From 2000 to 2015, the Pb release indicated a tendency to transfer from the middle east to the west, and the release center of Zn showed a trend of shifting from the south to north. Hunan Province showed the greatest release. The concentration of heavy metals in different regions displayed great differences and the single pollution indices of Cd, Pb, Cu, and Zn in Zhuzhou and Huludao all reached extremely polluted levels; however, in Tongguan, they were all below 1 and within the safe level. The potential ecological risk in Gejiu, Mianxian, Fengxian, Zhuzhou, Huludao, and Shaoguan was high, which indicated that there were very high potential ecological risks in those areas. Cd had the highest potential ecological risk in most smelting areas, followed by Pb. From the above analyses, it indicates that Pb/Zn smelting activities have caused soil heavy metal pollution to different degree. With environmental problems becoming more and more important, our study can provide some significant guidance for pollution control.

## Figures and Tables

**Table 1:** The P and Z values of Pb/Zn environmental release in China from the period 2000 to 2015.

	Year	Moran’s I	P-Value	E(I)	Z-Value
**Pb**	2000	0.0651	0.2252	−0.03125	1.2128
	2005	−0.0327	0.9834	−0.03125	−0.0209
	2010	−0.0109	0.7843	−0.03125	0.2737
	2015	−0.0118	0.7954	−0.03125	0.2593
**Zn**	2000	0.0686	0.2206	−0.03125	1.2249
	2005	0.1156	0.0637	−0.03125	1.8539
	2010	0.0152	0.5398	−0.03125	0.6131
	2015	0.0455	0.3514	−0.03125	0.9318

**Table 2:** The concentration of heavy metals in soil (mg/kg), (the concentration is shown as an average).

Region	N	Cd	Pb	Cu	Zn	Source
Huize, Yunnan	23	12.80	712.00	239.00	1688.00	[11]
Gejiu, Yunnan	15	63.50	3347.50	117.00	2974.30	[33]
Lancang, Yunnan	53	2.36	287.79	14.79	161.94	[34]
Hezhang, Guizhou	8	43.00	9000.00	120.00	11,000.00	[35]
Bijie, Guizhou	101	2.07	87.18	97.81	261.00	[12]
Baiyin ^a^, Gansu	33	15.93	392.59	330.81	737.18	[36]
Baiyin ^b^, Gansu	16	19.70	3510.00	426.00	332.00	[37]
Huixian ^a^, Gansu	21	4.37	418.00	29.50	90.10	[38]
Huixian ^b^, Gansu	27	9.85	655.89	37.24	435.89	[39]
Huixian ^c^, Gansu	20	3.12	214.00	25.80	79.50	[40]
Mianxian, Shaanxi	17	39.74	165.08	52.05	1304.27	[41]
Fengxiang, Shaanxi	27	0.95	53.50	30.81	118.27	[42]
Tongguan, Shaanxi	18	0.26	94.63	26.10	68.60	[32]
Fengxian, Shaanxi	9	33.79	412.28	59.14	556.73	[43]
Zhuzhou, Hunan	10	108.18	7621.86	261.41	1249.20	[44]
Chenzhou, Hunan	35	2.80	226.50	60.50	347.10	[45]
Jiyuan, Henan	45	5.75	145.00	22.00	84.70	[46]
Jiaozuo, Henan	135	1.70	42.33	15.98	67.77	[47]
Huludao, Liaoning	113	60.94	443.10	247.80	4084.00	[48]
Youxi, Fujian	12	8.28	699.25	114.88	670.47	[49]
Shaoguan, Guangdong	13	25.61	1185.66	67.13	764.12	[50]

Note: ^a^, ^b^, and ^c^ represent the different smelting areas in the same district, N represents the number of soil samples.
